# Vma8p-GFP Fusions Can Be Functionally Incorporated into V-ATPase, Suggesting Structural Flexibility at the Top of V1

**DOI:** 10.3390/ijms12074693

**Published:** 2011-07-20

**Authors:** Szczepan Nowakowski, Dalibor Mijaljica, Mark Prescott, Rodney J. Devenish

**Affiliations:** Department of Biochemistry and Molecular Biology, and ARC Centre of Excellence in Structural and Functional Microbial Genomics, Monash University, Clayton campus, Victoria 3800, Australia; E-Mails: szczepan.nowakowski@eyeandear.org.au (S.N.); Dalibor.Mijaljica@monash.edu (D.M.); Mark.Prescott@monash.edu (M.P.)

**Keywords:** V-ATPase, Vma8p, yeast

## Abstract

The vacuolar ATPase (V-ATPase) complex of yeast (*Saccharomyces cerevisiae*) is comprised of two sectors, V_1_ (catalytic) and V_O_ (proton transfer). The hexameric (A_3_B_3_) cylinder of V_1_ has a central cavity that must accommodate at least part of the rotary stalk of V-ATPase, a key component of which is subunit D (Vma8p). Recent electron microscopy (EM) data for the prokaryote V-ATPase complex (*Thermus thermophilus*) suggest that subunit D penetrates deeply into the central cavity. The functional counterpart of subunit D in mitochondrial F_1_F_O_-ATP synthase, subunit γ, occupies almost the entire length of the central cavity. To test whether the structure of yeast Vma8p mirrors that of subunit γ, we probed the location of the C-terminus of Vma8p by attachment of a large protein adduct, green fluorescent protein (GFP). We found that truncated Vma8p proteins lacking up to 40 C-terminal residues fused to GFP can be incorporated into functional V-ATPase complexes, and are able to support cell growth under alkaline conditions. We conclude that large protein adducts can be accommodated at the top of the central cavity of V_1_ without compromising V-ATPase function, arguing for structural flexibility of the V_1_ sector.

## 1. Introduction

Vacuolar ATPase (V-ATPase) is a multi-subunit protein complex which generates a chemiosmotic proton gradient across membranes of cells and intracellular organelles, by coupling hydrolysis of ATP to proton pumping [1]. Thus, this complex is responsible primarily for the acidification of intracellular vesicles and the yeast vacuole (equivalent of a lysosome) [1,2]. In yeast, a functional V-ATPase complex is also required for cell growth under alkaline conditions [3,4]. Analogous to F_1_F_O_-ATP synthases the V-ATPase complex is considered to becomprised of two sectors, a catalytic head sector (V_1_) and a membrane-embedded proton transfer sector (V_0_). These two sectors are joined via peripheral stator stalks and a central rotary stalk [5–8]. A rotary mechanism of catalysis, like that used by members of the F_1_F_O_-ATPase family of enzymes, also applies in V-ATPase [9–11].

The overall structure of the enzyme is known from electron microscopy (EM) data complemented by homology comparisons with other ATPases, especially the well-characterized mitochondrial F_1_F_O_-ATP synthase (mtATPase) [7,12–16]. A partial crystal structure of the partial V_1_ sector from *Thermus thermophilus* demonstrated structural homology between the V-ATPases and ATP synthase enzymes. Thus the bulk of the catalytic sector is formed from three pairs of AB subunits forming a hexameric cylinder (A_3_B_3_) having a central cavity which is occupied by the rotary stalk [17,18]. However, the available data suggest that V-ATPase is more complicated than F_1_F_O_-ATPase, having three stator stalks and containing several additional components integrated into the basic rotary motor structure presumably involved in controlling activity of the complex [8,14–16].

A key subunit of the rotary stalk of V-ATPase is subunit D (Vma8p in yeast) [19], whose rotation, relative to an immobilized V_1_ sector, has been demonstrated in isolated complexes using fluorescence imaging [10,20]. The available biochemical and structural data indicate that subunit D occupies at least part of the central cavity of V_1_, with a portion extending below the bottom of V_1_, where it interacts with the second central rotor subunit, F [13,15,16,18,21–23].

The tertiary structure of Vma8p remains undetermined, but the protein is similar in size and has a similar predicted high α-helical content to the mtATPase subunit γ. Furthermore, mutations leading to the substitution of residues in regions of Vma8p corresponding to regions of mtATPase subunit γ, which are essential for modulation of catalytic subunit activity and which play a role in coupling enzyme activity, were found to be deleterious to the enzymatic activity of the V-ATPase [24]. The crystal structure of the central stalk of the prokaryotic V-ATPase shows the helical domains of subunit D extending towards the top of the V_1_ cavity [17]. These data indicate that Vma8p adopts a similar tertiary structure to mtATPase subunit γ as previously suggested [24,25]. Subunit γ has two large α-helical segments, connected by an intervening loop sequence, which turn on each other to extend almost the entire length of the central cavity of F_1_ [26,27]. Both the amino and carboxyl termini of subunit γ point towards the top of F_1_, with the loop sequence at the bottom. However, the available data show that the helical domains of Vma8p do not reach the top of the V_1_ central cavity.

We sought to probe the location of the C-terminus of Vma8p by means of attachment of a large protein adduct and determining the consequences for V-ATPase function using a fluorescent marker of V-ATPase activity in live cells and the effect on the growth phenotype. We have previously used a similar approach to investigate the environment at the top of the central cavity of yeast mtATPase, by fusing green fluorescent protein (GFP) to the C-terminus of subunit γ [28]. Yeast strains expressing a V-ATPase that is dysfunctional fail to assemble correctly, or are inherently unstable, exhibit a *vma* phenotype [29]. Such a phenotype renders yeast cells unable to grow at alkaline pH, thus any significant loss of function in V-ATPase should be reflected in standard growth assays. The function of V-ATPase complexes *in vivo* was demonstrated directly by quinacrine staining of vacuoles.

## 2. Results and Discussion

### 2.1. Experimental Strategy

The diameter of the central cavity of V_1_ has been estimated at 17–21 Å in the V-ATPase of bovine clathrin-coated vesicles [6,30]. A somewhat larger estimate of 29 Å has been obtained for the central cavity of the isolated V_1_ sector of the yeast V-ATPase [13], although this may reflect dynamic changes caused as a result of dissociation of V_1_ from the V_O_ sector, or differences in experimental conditions [31]. These results are consistent also with the structure of the A_3_B_3_ central cavity as determined for *T. thermophilus* [17].

Our experimental strategy assumes the C-terminus of Vma8p is located within the central cavity of V_1_. In its native fluorescent state, GFP assumes a very stable, three-dimensional structure [32], 48 Å in length and 24 Å in diameter. When fused to the C-terminal end of Vma8p, the presence of a correctly folded GFP moiety within the confines of the central cavity of V_1_ would likely compromise V-ATPase function by steric hindrance of other protein subunits comprising V_1_. We have previously demonstrated for the yeast mtATPase complex that bringing a GFP molecule in close proximity to the top of the central cavity of F_1_, by fusing it directly to the γ subunit, has a detrimental effect on enzyme activity [28]. Moreover, the fact that GFP must assume its native conformational state in order to fluoresce provides a convenient *in vivo* reporter of the tertiary structural state of the GFP molecule, as well as its localization within the cell. To increase the likelihood of expressing a fusion product presumed to be incapable of being accommodated with the central cavity of V_1_ we constructed two fusion proteins where the GFP molecule was added to the end of a Vma8p protein in which either 40 or 47 C-terminal amino acids were removed.

### 2.2. Truncated Vma8p Variants Tagged with GFP are Functional

Strains were constructed by genomic integration in which the endogenous VMA8 ORF, encoding native Vma8p, was replaced by an expression cassette containing an ORF encoding truncated versions of Vma8p as C-terminal fusions with GFP. To determine whether any growth phenotype observed resulted from the presence of the GFP moiety, and not on the removal of essential portions of the Vma8p C-terminus, we constructed corresponding strains in which the truncated Vma8p proteins were not fused to GFP. The strains constructed are summarized in Table 1.

**Figure 1:**
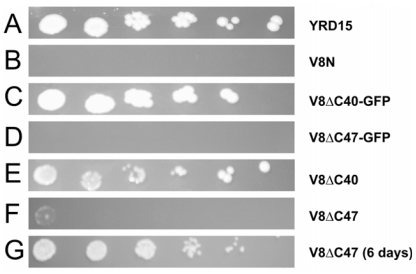
Growth characteristics of strains. Strains tested expressed Vma8p–GFP (**C**–**D**) fusion proteins, or truncated versions of Vma8p (**E**–**G**) and were compared to parental strain YRD15 (**A**) and a *vma8* null strain V8N (**B**). All strains were grown overnight in liquid YEPD medium. Cell density was adjusted to 5 × 10^6^ cells/mL, then samples of each culture were serially diluted five-fold and 2 μL aliquots of each dilution dropped out onto YEPD plates buffered to pH 7.5. Plates were incubated at 28 °C for 3 days. Strains which had failed to grow after 3 days were incubated for a further 3 days.

To establish whether the expressed fusion proteins were able to functionally substitute for the native Vma8p protein without compromising the function of the V-ATPase, growth was assayed under alkaline pH conditions (Figure 1).

All strains grew equally well on YEPD plates at pH 5.5 (data not shown). As expected strain V8N did not grow at pH 7.5 (Figure 1B), due to its inability to form a functional V-ATPase complex. Growth comparable to that of the parental wild-type strain YRD15 (Figure 1A) was observed for the strain expressing GFP tagged to Vma8p truncated by 40 amino acids at the C-terminus (Figure 1C). By contrast, the truncation variant lacking 47 amino acids fused with GFP resulted in a *vma* phenotype (Figure 1D). The strain expressing the truncated version of Vma8p with 40 amino acids removed and not fused to GFP showed a wild-type phenotype growth (Figure 1E). Strain V8ΔC47 was also able to grow at pH 7.5, albeit more slowly than the wild-type strain (comparable growth after six days compared to three days for the wild-type; Figure 1F–G). These results indicate that the fusion protein expressed in the strain V8ΔC47-GFP affected the ability of V-ATPase to function normally.

If the C terminus of Vma8p faces towards the top of the V_1_ central cavity, these results would require the GFP to be accommodated within the upper part of the central cavity of V_1_. Taking into account the structural data placing the C terminal domain of subunit D within the central cavity [8,17], the GFP moiety of our fusion proteins appears to be accommodated within the central cavity of V_1_ without disrupting the function of the enzyme. Additionally, substitutions in the C-terminal region Lys^209^–Met^221^ of Vma8p have been demonstrated to cause loss of enzymatic activity and uncoupling of the V-ATPase, in some cases sufficient to produce a classical *vma* phenotype [24]. However, our data indicate that an active enzyme capable of supporting growth at pH 7.5 can be assembled using a truncated version of Vma8p (Met^1^–Lys^209^) lacking this region. This suggests that the substitutions arising as a consequence of mutations introduced into the Lys^209^–Met^221^ region impeded the function of the enzyme by steric hindrance rather than by removal of essential amino acid residues.

### 2.3. The Vma8p-GFP Fusion Proteins are Expressed Intact

**Figure 2:**
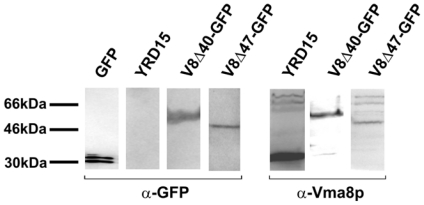
Expression of Vma8p-GFP fusion proteins. Yeast cultures were grown in liquid YEPD medium buffered to pH 7.5, with the exception of V8ΔC47-GFP which was grown in YEPD medium buffered to pH 5.5. Protein lysates of cells were subjected to SDS-PAGE under reducing conditions. Gels were transferred to PVDF membrane and probed with antibodies against GFP (monoclonal) or Vma8p (polyclonal) as indicated below the membrane strips. Blots were developed with Vistra ECF substrate and visualized using a Wallac Storm chemifluorescence scanner. Rainbow^TM^ Marker protein size standards are shown at left. A control lane containing unfused GFP purified after expression in bacterial cells has been included at left.

To determine the integrity of the fusion proteins within cells, whole cell protein extracts were prepared from each strain and proteins subjected to SDS-PAGE followed by transfer to PVDF membrane. Duplicate membranes were probed with antibody against either GFP or Vma8p (Figure 2).

The blots showed single polypeptide bands in the range of 50 to 60 kDa (Figure 2, left panel strips, probed with antiserum against GFP; right panel strips, probed with polyclonal antiserum against Vma8p), corresponding to the sizes expected for intact, uncleaved fusion proteins of Vma8p with GFP (Table 1). Furthermore, the absence of any bands corresponding to free GFP on the blots indicated GFP within cells was fused to Vma8p, and that the fusion proteins were not degraded or proteolytically digested in such a way as to release free GFP. Thus, no evidence of degradation of the fusion proteins was seen and these data indicate that the ability of strains expressing tagged Vma8p constructs to grow does not arise from loss of the fluorescent tag from the fusion protein.

### 2.4. Fluorescence Signals at the Vacuolar Membrane Indicate that the Fusion Proteins are Correctly Targeted and Folded

**Figure 3:**
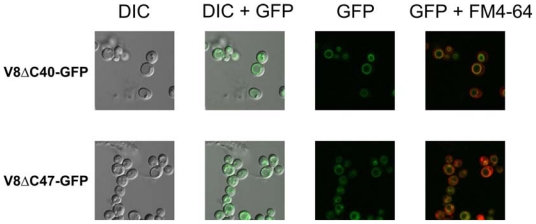
The fluorescence signal from GFP localizes to the vacuolar membrane in strains expressing Vma8p-GFP fusion proteins. Samples of cells grown in liquid YEPD were taken during the exponential phase of growth and imaged using a Fluoview FV500 Confocal Laser Scanning Microscope (Olympus, Australia) for either DIC or fluorescence. Cells were treated with FM 4-64, a dye that specifically labels the outer leaflet of the vacuolar membrane, and the fluorescence signal imaged and overlayed with the fluorescence signal for GFP [33].

The assembly of the fusion proteins into V-ATPase complexes on the vacuolar membrane was demonstrated by fluorescence microscopy of live yeast cells (Figure 3).

Cells showing a ring of green fluorescence co-localizing with the vacuolar membrane were observed for the strains expressing a Vma8p-GFP fusion protein (Figure 3). These results demonstrate that the fusion proteins were correctly targeted to assembled complexes on the vacuolar membrane. The fluorescence signals observed also indicated that the GFP molecules associated with the vacuolar membrane were capable of folding correctly into their native state.

Reversible disassembly of the V-ATPase has been demonstrated in yeast when cells are shifted from a glucose-rich medium to medium lacking glucose, with the V_1_ sector detaching from the vacuolar membrane [34]. When the strains expressing the Vma8p-GFP fusion proteins were shifted to a growth medium containing ethanol instead of glucose as the sole carbon source, the pattern of the fluorescence signal shifted from a concentrated ring around the vacuolar membrane to a diffuse cytosolic fluorescence (data not shown). This result provides additional evidence that the GFP tagged subunit is incorporated into properly assembled and functional V-ATPase complexes.

### 2.5. Vacuolar Uptake of Quinacrine *in Vivo* Indicates that V-ATPase Complexes Incorporating Vma8p-GFP Fusions are Functional

**Figure 4:**
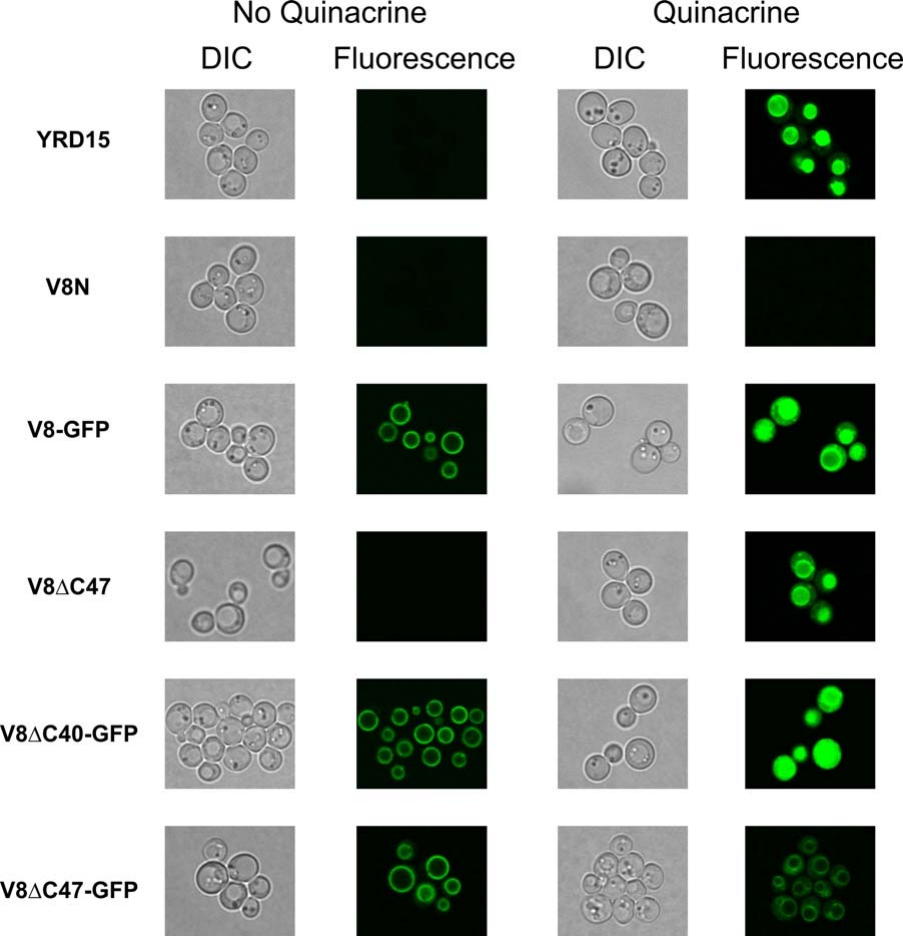
The fluorescence signal from quinacrine localizes to the vacuolar lumen in YRD15 (wild-type) strain as well as in strains V8-GFP (expressing the full length Vma8p protein fused at its C-terminus to GFP), V8ΔC47 and V8ΔC40-GFP, but not in *vma8* null strain (V8N) and V8ΔC47-GFP.

To demonstrate that V-ATPase complexes incorporating the fusion proteins were functional *in vivo*, the strains were incubated with quinacrine (a weakly basic dye which accumulates in low pH-compartments, such as the vacuole) [35], and examined by fluorescence microscopy (Figure 4). The uptake of quinacrine into vacuoles indicates that the V-ATPase complexes are functioning correctly. Consistent with the growth assays, the wild-type strain YRD15, strain expressing truncated Vma8p (lacking 47 C-terminal residues, V8ΔC47), and the strain expressing the V8ΔC40-GFP fusion demonstrated quinacrine uptake (Figure 4). This assay indicates that V-ATPase complexes incorporating the V8ΔC40-GFP fusion are functional *in vivo*. In contrast, incorporation of the truncated variant lacking the 47 C-terminus residues of Vma8p fused to GFP (V8ΔC47-GFP) leads to an inactive V-ATPase complex, indicating that the GFP moiety of the fusion protein, and not the lack of the Vma8p 47 C-terminal residues *per se*, causes impairment of V-ATPase function.

### 2.6. How Can a Vma8p-GFP Fusion Protein be Incorporated into Functional V-ATPase Complexes?

The results indicate that V-ATPase complexes incorporating Vma8p proteins in which up to 47 of the C-terminal residues have been truncated can retain function. Strains expressing fusion proteins in which GFP is attached at the C-terminus of a truncated Vma8p where up to 40 amino acid residues from the Vma8p C-terminus are missing, also exhibit a wild-type phenotype, and have functional V-ATPase complexes as demonstrated by *in vivo* quinacrine staining. Based on current structural data for V-ATPase [8,17,18], where the C terminal domain of Vma8p is located within the V_1_ central cavity, with its terminal residue facing toward the top of the cavity, we propose that our fusion proteins assemble into V-ATPase complexes in such a way that the GFP moiety is accommodated within the central cavity of V_1_. Given the limited space within the cavity, and the large tertiary structure of GFP, it is likely that the presence of a GFP molecule would alter the structure of the V_1_ sector. The strain expressing Vma8p lacking 47 C-terminal residues and fused to GFP, fails to assemble a functional V-ATPase complex, as demonstrated by growth assays and *in vivo* quinacrine staining. Presumably this fusion protein represents a point at which the GFP moiety is brought far enough into the central cavity of V_1_ that it interferes with, or distorts the structure of V_1_ essential for function. The introduction of a correctly folded GFP moiety into the limited space of the central cavity of V_1_ suggests that the top of the V_1_ sector may possess a significant degree of structural flexibility. The failure of complexes incorporating the Vma8p-GFP fusion protein where 47 C-terminal residues of Vma8p are missing, indicates that this flexibility is limited. Another possibility is that enzyme function is compromised by steric hindrance of key residues of the A and B subunits that face into the central cavity at the top of V_1_, suggesting that they may be involved in coupling of ATP hydrolysis to the rotary mechanism of the enzyme. Further structural data would be required to more fully appreciate these results.

## 3. Experimental Section

### 3.1. Materials

All materials, unless otherwise noted, were purchased from Sigma. Antiserum against Vma8p was a kind gift from Tom Stevens (University of Oregon). Antisera against GFP, and complete protease inhibitors were obtained from Boehringer Mannheim (Sydney, Australia). Anti-(mouse IgG) and anti-(rabbit-IgG), and Vistra ECF substrate were purchased from GE Healthcare Bio-sciences (Australia).

### 3.2. Yeast Strains and Growth Media

The parental strain for this study was YRD15 (MAT-α, *his3*, *leu2*, *ura3* [rho^+^]) [36] from which all strains expressing a Vma8p variant were derived. In these strains the native *VMA8* gene, expressing wild-type Vma8p, was modified by a PCR-based integration method, essentially as described previously [37], to produce a series of strains expressing truncated Vma8p variants either fused or not to a fluorescent protein (see Table 1). The template for PCR amplifications was the plasmid pAS1NB: YEGFP_ADHT_HIS3 [38]. High efficiency transformation of PCR products into yeast cells was performed using the lithium acetate/PEG method of Gietz and Woods [39]. Transformants expressing the GFP fusions were selected for growth on medium lacking histidine. Strain KHY104 (MAT-α, *ade6, his4-519*, *leu2-3,112*, *ura3-52*, *pep4-3, gal2 vma8Δ::LEU2*) [19], which does not express Vma8p, is designated here as V8N and was used as a control strain in comparative growth and microscopy assays.

### 3.3. Testing the Growth of Yeast Strains Expressing Vma8p Variants

Cells were grown in liquid YEPD medium overnight at 28 °C, with rotation. Equal cell numbers of each strain were serially diluted and spotted onto solid YEPD medium containing 50 mM MES/MOPS and buffered to either pH 5.5 or 7.5. Plates were incubated at 28 °C for 3–6 days before scoring.

### 3.4. Whole Cell Protein Extraction

Cells were grown in liquid YEPD medium buffered to pH 5.5 or 7.5. Proteins were extracted from 5 mg of cells collected during the mid-logarithmic phase of growth as described by Yaffe [40].

### 3.5. Gel Electrophoresis and Western Blotting

SDS-PAGE was carried out using pre-cast 4–20% gradient acrylamide gels. Rainbow^TM^ Markers (GE Healthcare Bio-sciences, Australia) were used as molecular weight standards. Proteins were transferred to PVDF membrane (Gelman Laboratory, Pall Corporation) using standard procedures. The membranes were probed with either a mouse monoclonal antiserum against GFP (diluted to 1:1000) or a rabbit polyclonal antiserum against Vma8p (diluted to 1:200). Secondary antibodies used were alkaline phosphate-conjugated anti-mouse and anti-rabbit IgG. The blots were then washed and signals were generated by overlaying the membrane with chemi-fluorescent Vistra ECF substrate. Membranes were then scanned using a Storm 860 Phosphoimager (Molecular Dynamics).

### 3.6. Fluorescence Microscopy

Fluorescence microscopy was performed using a Fluoview FV500 Confocal Laser Scanning Microscope (Olympus, Australia).

### 3.7. Quinacrine Staining

Quinacrine staining was performed using the technique described by Roberts *et al*. [35]. Samples of cell cultures grown in liquid YEPD were sampled during the exponential phase of growth and transferred into a microcentrifuge tube containing 200 μM quinacrine and incubated for 5 min at room temperature. Cells were harvested by a 30 sec centrifugation and washed once in the YEPD medium. Finally, cells were resuspended in 100 μL of YEPD medium and then 10 μL was spotted onto a microscope slide containing 20 μL of low melting agarose. Fluorescence microscopy was used to visualize the uptake of quinacrine.

## 4. Conclusions

We have shown that truncated Vma8p proteins lacking up to 40 C-terminal residues, fused to GFP, can be incorporated into functional V-ATPase complexes, as demonstrated by the growth of cells under alkaline conditions, localization of the fluorescent label with the vacuolar membrane, and quinacrine labeling *in vivo*. Based on current structural data for the V-ATPase, we conclude that the Vma8p-GFP fusion proteins are accommodated within the central cavity of V_1_, arguing for structural flexibility of the enzyme, similar to that previously demonstrated at the top of F_1_ for mtATPase [27].

## Figures and Tables

**Table 1:** Strains used in this study.

Strain	Vma8p variant expressed	Molecular Mass (kDa)
YRD15 (wild-type)	Full Vma8p (residues 1-256)	29.2
V8N	No Vma8p expressed (gene deleted)	0
V8ΔC40-GFP	Residues 1 to 216 of Vma8p + GFP	51.6
V8ΔC47-GFP	Residues 1 to 209 of Vma8p + GFP	50.8
V8ΔC40	Residues 1 to 216 of Vma8p	24.7
V8ΔC47	Residues 1 to 209 of Vma8p	23.9
